# Robot-Assisted Laparoscopic Radical Prostatectomy for Prostatic Metastatic Recurrence from Testicular Cancer

**DOI:** 10.1155/2024/1941414

**Published:** 2024-06-11

**Authors:** Kohei Hirose, Yasukazu Nakanishi, Ryo Andy Ogasawara, Naoki Imasato, Sao Katsumura, Madoka Kataoka, Shugo Yajima, Hitoshi Masuda

**Affiliations:** National Cancer Center Hospital East, Kashiwa City, Chiba, Japan

## Abstract

**Introduction:**

Treatment evidence for malignancies metastatic to the prostate in young patients is scarce. Herein, we present a case of prostatic metastasis from testicular cancer treated with induction chemotherapy followed by robot-assisted radical prostatectomy. *Case Presentation.* The patient is a 34-year-old male who underwent radical orchiectomy for a left testicular tumor two years ago and was diagnosed with a mixed germ cell tumor. He was followed up without adjuvant therapy, but symptoms of dysuria lead to suspicion of a prostate tumor, which was diagnosed by prostate biopsy as seminoma of the prostate. After four cycles of chemotherapy, normalization of tumor markers, and tumor shrinkage on imaging, he underwent robot-assisted radical prostatectomy. No recurrence has been observed nine months after treatment.

**Conclusion:**

In men with a history of testicular cancer presenting with lower urinary tract symptoms, it is important to consider recurrent prostate metastases.

## 1. Introduction

Testicular cancer is the most common solid tumor in adolescent young adult men and can be categorized as seminoma and nonseminoma [1]. Nonseminoma is the more aggressive tumor subtype; however, in recent years, its prognosis has been generally good, with 5-year survival rates of up to 70%, even in cases of advanced/metastatic cancer [2].

Following chemotherapy, surgical resection of the residual masses is recommended when tumor marker levels normalize [3]. However, there is no clear evidence regarding the need for surgical consolidation after chemotherapy at sites outside the retroperitoneum.

In testicular cancer, the primary metastatic organs are the lungs, distant lymph nodes, and liver [4]. Prostate metastasis of testicular cancer is very rare, with 10 cases reported to date. Herein, we report a case of metastatic recurrence of testicular cancer in the prostate that was treated with induction chemotherapy followed by robot-assisted radical prostatectomy (RARP).

## 2. Case Presentation

The patient was a 34-year-old man who had initially undergone a left radical orchiectomy for a left testicular tumor 2 years ago. He had no relevant medical or family histories. Pathological examination revealed a mixed germ cell tumor consisting of embryonal carcinoma and seminoma with syncytiotrophoblastic giant cells (pT2, with vascular invasion). Alpha-fetoprotein (AFP), *β*-human chorionic gonadotrophin (*β*-HCG), and lactate dehydrogenase (LDH) levels were within normal ranges, and the diagnosis was stage IB nonseminomatous germ cell tumor. Postoperative adjuvant chemotherapy was recommended; the patient selected the surveillance rather than adjuvant chemotherapy. He was followed up with regular tumor marker tests and computed tomography (CT) scans.

**Figure 1:**
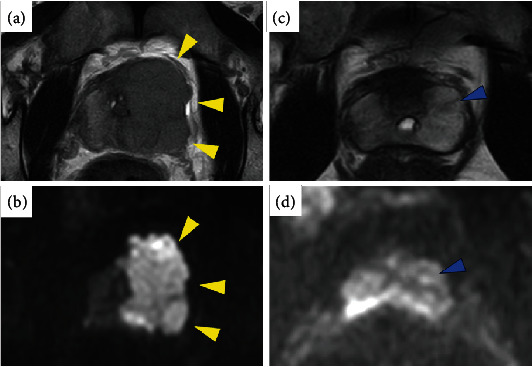
Pelvic magnetic resonance imaging: (a) axial T2-weighted image and (b) diffusion-weighted image revealed prostate tumor at diagnosis of metastasis. (c) Induction chemotherapy resulted in prominent shrinkage of the tumor and (d) obscuration of the high signal on diffusion-weighted image.

Subsequently, the patient presented with dysuria. Digital rectal examination revealed an elastic hard mass around the prostate, and pelvic magnetic resonance imaging (MRI) showed a prostatic mass invading the capsule, seminal vesicle, and membranous urethra (Figures 1(a) and 1(b)). Tumor markers at that time were as follows: AFP 4.5 ng/ml, *β*-HCG 5.2 mIU/ml, LDH 429 U/L, and PSA 7.56 ng/ml, respectively. Contrast-enhanced CT revealed no obvious lymph node enlargement or evidence of visceral metastases other than those in the prostate. He underwent a transperineal prostate biopsy, which showed a seminoma component in all eight cores (HCG[-], CD30[+], c-kit[+], D2-40[+], SALL[+], and prostate-specific antigen [PSA][-]). The patient was then referred to our hospital.

The diagnosis was prostate metastatic recurrence from testicular cancer, and the patient was considered at risk of a poor prognosis according to the International Germ Cell Classification [5]. We planned four cycles of bleomycin-etoposide and platinum chemotherapy followed by RARP. As a result of chemotherapy performed after sperm cryopreservation, all tumor markers normalized, and CT and MRI showed a prominent reduction in prostate metastasis (Figures 1(c) and 1(d)). However, residual disease after chemotherapy could not be completely ruled out using imaging, and prostate rebiopsy might show false-negative results. Therefore, the patient and the surgeon prioritized radical prostatectomy. The operative time was 111 min, and the estimated blood loss was clinically insignificant (less than 50 ml). A pelvic lymph node dissection or nerve-sparing surgery was not performed. The patient was discharged 7 days postoperatively with no apparent complications. Pathological examination revealed no residual tumors.

Twelve months postoperatively, the patient showed no clinical, laboratory, or imaging findings indicating recurrence. Regarding his urinary continence, he has also been pad-free since 1 month postoperatively. We assessed the International Index of Erectile Function (IIEF-5) [6] and found a score of 7 at the 12-month follow-up.

## 3. Discussion

To our knowledge, only 10 cases of metastatic testicular cancer recurrence in the prostate have been reported (Table 1) [7–15]. Five of the cases had late recurrence > 10 years after radical orchiectomy and postoperative adjuvant treatment, and all were detected by physical symptoms. Prostate metastatic recurrence has also been reported in stage 1 testicular tumors through surveillance, radiotherapy, or retroperitoneal lymph node dissection.

One case of testicular cancer with prostate metastasis at initial presentation has been reported [16]. However, prostate metastatic recurrence in patients with stage 1 testicular tumors treated with adjuvant chemotherapy following radical orchiectomy has not been reported. This indicates that micrometastasis of testicular cancer to the prostate is not very rare and that many of these lesions may go into remission without manifestation once chemotherapy is administered. The mechanism of testicular cancer metastasis to the prostate is unclear; however, Torelli et al. suggested that lymphatic rather than hematogenous metastasis is more likely given the paucity of reports on metastasis to other organs [11]. Sagalowsky et al. also mentioned the possibility of metastasis via the spermatic cord [7], which is corroborated by a small number of reports on testicular metastasis in prostate cancer [17, 18]. Since our patient presented with solitary prostate recurrence and there was no residual disease or obvious localization of necrotic tissue in the resected specimen, speculating on the mechanism of metastasis was difficult.

Herein, the patient was young. Although induction chemotherapy, including cisplatin, has been associated with a temporary decrease in fertility [18], the loss of ejaculation due to radical prostatectomy was definite in this case. Given the good response to induction chemotherapy as previously reported, the indications for surgical resection of the prostate should be carefully considered in terms of the preservation of fertility and urinary function. In the present case, residual disease after induction chemotherapy could not be completely ruled out by imaging. On the other hand, prostate rebiopsy might show false-negative results and was not performed in two previously reported cases in which surgical resection was performed for prostate metastases [9, 12]. We discussed the treatment strategy with an in-house cancer board consisting of urologists, medical oncologists, radiologists, and pathologists. As a consolidation therapy, we also considered that radiotherapy could be alternative to surgery while preserving sexual function. However, due to insufficient evidence for radiotherapy for residual tumor of nonseminoma, the overall consensus was to recommend surgery. Hence, we made the decision for radical prostatectomy. We have suggested him the option of performing a saturation prostate biopsy prior to RARP. However, he was also convinced to undergo the surgery due to the possibility of false-negative biopsy results. Therefore, the patient and the surgeon prioritized surgery.

Prostate lesions are likely to be undetectable on CT scans, which are usually performed at follow-up because of the limitations of contrast resolution. Although rare, when a patient with a history of testicular cancer presents with lower urinary tract symptoms, the possibility of prostatic metastasis should be considered.

## 4. Conclusion

The possibility of metastatic cancer recurrence in the prostate should be considered when a man with a history of testicular cancer presents with lower urinary tract symptoms.

RARP after induction chemotherapy for prostate metastatic recurrence of testicular cancer may be a useful option for surgical consolidation. The indications for this procedure, which can affect sexual and urinary functions, should be carefully considered in anticipation of a favorable response to induction chemotherapy.

## Figures and Tables

**Table 1:** Summary of the case reports of prostate metastases from testicular cancer in the literature.

No.	Author (year)	Pathology of primary site	Stage	Adjuvant therapy	Response	Time to recurrence (mo.)	Reason for inspection	Recurrence site	Pathology of recurrence site	Initial therapy for recurrence	Response to initial therapy	Adjuvant therapy for recurrence	Follow-up (mo.)
1	Sagalowsky (1986) [7]	Seminoma	I	None	—	24	Symptoms	Prostate, retroperitoneal lymph nodes	Seminoma	BEP^∗^3	PR	RT	24
2	Plummer (2000) [8]	Seminoma	I	RT	—	24	Tumor marker	Prostate, lung, iliac lymph nodes	Seminoma	BEP^∗^6	CR	None	48
3	Farnham (2005) [9]	Seminoma	IV	CT (BEP^∗^4)	CR	36	Symptoms	Prostate	Seminoma	VIP^∗^4	PR	Cystoprostatectomy	48
4	Alsharif (2008) [10]	Seminoma	Unknown	RPLND	—	180	Symptoms	Prostate	Seminoma	EP^∗^4	CR	None	12
5	Torelli (2013) [11]	Embryonal carcinoma + mature teratoma + seminoma	I	RPLND	—	36	Tumor marker	Prostate, pelvic lymph nodes	Seminoma	BEP∗3	PR	RPLND, VIP^∗^4, RT	78
6	Torelli (2013) [11]	Embryonal carcinoma + mature and immature teratoma	I	RPLND	—	252	Symptoms	Prostate, pelvic lymph nodes	Nonseminoma (no detail)	BEP^∗^4	PR	PLND	69
7	Janowitz (2015) [12]	York sac tumor	I	None	—	264	Symptoms	Prostate, lung, pelvic lymph nodes	York sac tumor	BEP^∗^2 + VIP ^∗^2	PR	Cystoprostatectomy	36
8	Baunacke (2015) [13]	Seminoma	I	RT	—	120	Symptoms	Prostate	Undifferentiated sarcomatoid tumor	Prostatectomy + PLND	—	None	6
9	Durer (2019) [14]	Seminoma	I	None	—	36	Imaging	Prostate, pelvic lymph nodes	Seminoma	BEP^∗^unknown	CR	None	2
10	Baweja (2022) [15]	Seminoma	I	RT	—	240	Symptoms	Prostate, pelvic lymph nodes	Seminoma	EP^∗^4	CR	None	24
11	Present report (2023)	Embryonal carcinoma + seminoma	l	None	—	27	Symptoms	Prostate	Seminoma	BEP^∗^4	CR	Prostatectomy	9

CT = chemotherapy; RPLND = retroperitoneal lymph node dissection; PLND = pelvic lymph node dissection; BEP = bleomycin+etoposide+cisplatin; VIP = etoposide+ifosfamide+ bleomycin; EP = etoposide+cisplatin; RT = radiotherapy; CR = complete remission; PR = partial remission.

## Data Availability

The data that support the findings of this study are available on request from the corresponding author. The data are not publicly available due to restrictions on their containing information that could compromise the privacy of research participants.

## References

[1] Pishgar F., Haj-Mirzaian A., Ebrahimi H. (2019). Global, regional and national burden of testicular cancer, 1990-2016: results from the global burden of disease study 2016. *BJU International*.

[2] Gilligan T., Lin D. W., Aggarwal R. (2019). Testicular cancer, version 2.2020, NCCN clinical practice guidelines in oncology. *Journal of the National Comprehensive Cancer Network*.

[3] Riggs S. B., Burgess E. F., Gaston K. E., Merwarth C. A., Raghavan D. (2014). Postchemotherapy surgery for germ cell tumors--what have we learned in 35 years?. *The Oncologist*.

[4] Xu P., Wang J., Abudurexiti M. (2020). Prognosis of patients with testicular carcinoma is dependent on metastatic site. *Frontiers in Oncology*.

[5] Wilkinson P. M., Read G. (1997). International Germ Cell Consensus Classification: a prognostic factor-based staging system for metastatic germ cell cancers. International germ cell cancer collaborative group. *Journal of Clinical Oncology*.

[6] Rhoden E. L., Telöken C., Sogari P. R., Vargas Souto C. A. (2002). The use of the simplified international index of erectile function (IIEF-5) as a diagnostic tool to study the prevalence of erectile dysfunction. *International Journal of Impotence Research*.

[7] Sagalowsky A. I., McConnell J. D., Admire R. (1986). Uncommon sites of recurrent seminoma and implications for therapy. *Cancer*.

[8] Plummer E. R., Greene D. R., Roberts J. T. (2000). Seminoma metastatic to the prostate resulting in a rectovesical fistula. *Clinical Oncology*.

[9] Farnham S. B., Mason S. E., Smith J. A. (2005). Metastatic testicular seminoma to the prostate. *Urology*.

[10] Alsharif M., Aslan D. L., Jessurun J., Gulbahce H. E., Pambuccian S. E. (2008). Cytologic diagnosis of metastatic seminoma to the prostate and urinary bladder: a case report. *Diagnostic Cytopathology*.

[11] Torelli T., Lughezzani G., Catanzaro M. (2013). Prostatic metastases from testicular nonseminomatous germ cell cancer: two case reports and a review of the literature. *Tumori*.

[12] Janowitz T., Welsh S., Warren A. Y. (2015). Prostatic relapse of an undifferentiated teratoma 24 years after orchidectomy. *BMC Research Notes*.

[13] Baunacke M., Toma M., Novotny V., Fröhner M., Wirth M., Huber J. (2015). Incidental undifferentiated carcinoma of the prostate: a case with unusual diagnosis. *Der Urologe*.

[14] Durer C., Comba I. Y., Durer S., Torres Luna N., Jignesh P., Carilli A. (2020). Seminoma metastasized to the prostate: a case report and literature review. *Urology Case Reports*.

[15] Baweja A., Mar N., Rezazadeh K. A. (2022). Late recurrence of localized pure seminoma in prostate gland: a case report. *World Journal of Clinical Oncology*.

[16] Noda T., Fujisaki A., Uchida K. (2023). A case of prostatic metastasis from non-seminomatous testicular cancer. *IJU Case Reports*.

[17] Hong J. H., Nindra U., Nguyen R., Gassner P., Balakrishnar B., Rutland T. (2022). A rare case of castrate-resistant prostate adenocarcinoma with a unilateral testicular metastasis mimicking a primary testicular tumour. *Case Reports in Oncology*.

[18] Howell S. J., Shalet S. M. (2001). Testicular function following chemotherapy. *Human Reproduction Update*.

